# Evaluation of Syrosingopine, an MCT Inhibitor, as Potential Modulator of Tumor Metabolism and Extracellular Acidification

**DOI:** 10.3390/metabo12060557

**Published:** 2022-06-17

**Authors:** Chloe Buyse, Nicolas Joudiou, Aude Warscotte, Elena Richiardone, Lionel Mignion, Cyril Corbet, Bernard Gallez

**Affiliations:** 1 Louvain Drug Research Institute, Biomedical Magnetic Resonance (REMA), Université Catholique de Louvain (UCLouvain), 1200 Brussels, Belgium; chloe.buyse@uclouvain.be (C.B.); aude.warscotte@student.uclouvain.be (A.W.); lionel.mignion@uclouvain.be (L.M.); 2 Louvain Drug Research Institute, Nuclear and Electron Spin Technologies (NEST) Platform, Université Catholique de Louvain (UCLouvain), 1200 Brussels, Belgium; nicolas.joudiou@uclouvain.be; 3 Pole of Pharmacology and Therapeutics (FATH), Institut de Recherche Expérimentale et Clinique (IREC), Université Catholique de Louvain (UCLouvain), 1200 Brussels, Belgium; elena.richiardone@uclouvain.be (E.R.); cyril.corbet@uclouvain.be (C.C.)

**Keywords:** tumor metabolism, pHe, proliferation, MCT, CEST-MRI, syrosingopine

## Abstract

Extracellular acidification has been shown to be an important characteristic of invasive tumors, as it promotes invasion and migration but also resistance to treatments. Targeting transporters involved in the regulation of tumor pH constitutes a promising anti-tumor approach, as it would disrupt cellular pH homeostasis and negatively impact tumor growth. In this study, we evaluated the impact of syrosingopine, an inhibitor of MCT1 and MCT4, as a modulator of tumor metabolism and extracellular acidification in human breast cancer (MDA-MB-231) and pharyngeal squamous cell carcinoma (FaDu) cell models. In both models in vitro, we observed that exposure to syrosingopine led to a decrease in the extracellular acidification rate, intracellular pH, glucose consumption, lactate secretion and tumor cell proliferation with an increase in the number of late apoptotic/necrotic cells. However, in vivo experiments using the MDA-MB-231 model treated with a daily injection of syrosingopine did not reveal any significant change in extracellular pH (pHe) (as measured using CEST-MRI) or primary tumor growth. Overall, our study suggests that targeting MCT could lead to profound changes in tumor cell metabolism and proliferation, and it warrants further research to identify candidates without off-target effects.

## 1. Introduction

Altered glucose metabolism is considered a hallmark of cancer [1]. Elevated glycolysis and lactate production are often found in tumor cells and persist even under normoxic conditions [2]. This upregulated glucose consumption leads to the production of a large amount of lactate and protons, which can result in extracellular acidosis when wash-out of the latter is impaired due to a dysfunctional tumor vasculature [3]. Extracellular acidification has been shown to be an important characteristic of invasive tumors, as it promotes invasion and migration but also resistance to treatments [4,5,6]. As variations in intracellular pH (pHi) can influence the ionization and function of diverse proteins, ultimately leading to cell death, cells need to fight against intracellular acidification [7]. Several transporters and proteins are involved in the regulation of tumor pH. Their expression and/or activity are increased by intracellular acidification and the activation of hypoxia-inducible factor-1 (HIF-1) [7]. The most commonly described pH regulators include carbonic anhydrases 2, 9 and 12 (CAII, CAIX and CAXII, respectively); Na^+^/H^+^ exchanger 1 (NHE1); Na^+^/HCO_3_^−^ co-transporters (NBCs); and monocarboxylate transporters MCT1, MCT2, MCT3 and MCT4 [8]. Targeting one or more of these transporters constitutes a promising anti-tumor approach, as it would disrupt pH homeostasis and possibly negatively impact tumor growth [8]. Preclinical and some clinical studies have shown that targeting acidity can improve therapeutic responses [9]. Proton-pump inhibitors (PPIs), such as omeprazole, are well-tolerated antiacids and have been used to decrease tumor acidity, suppress tumor growth in mouse models and overcome acid-related chemoresistance [7,10,11]. Targeting CAIX has also received consideration because CAIX is usually overexpressed in the majority of solid tumors. Studies have shown that CAIX inhibition might reduce tumor cell proliferation, invasion and migration [12].

Monocarboxylate transporters are lactate/H^+^ symporters, playing a major role in H^+^ and lactate efflux and pH homeostasis regulation. There are 14 identified MCT isoforms, with two of them, MCT1 and MCT4, being associated with cancer aggressiveness and poor prognosis [13,14]. MCT1 and MCT4 are important actors in the metabolic symbiosis between glycolytic and oxidative tumor cells, where both cell types rely on each other to regulate their energy metabolites [15]. Sonveaux et al. [15] showed that MCT1 inhibition led to a metabolic switch from lactate-fueled respiration to glycolysis, induced cell death in hypoxic/glycolytic tumor cells and increased tumor radiosensitization. The pharmacological or genetic ablation of MCT1 or MCT4 activity led to reduced proliferation in vitro and in vivo [16]. Benjamin et al. [17,18] reported that lactate and H^+^ efflux could be inhibited by syrosingopine. Syrosingopine is an antihypertensive reserpine derivative, also known to inhibit MCT1 and MCT4, with a 60-fold higher potency on MCT4. They found that syrosingopine, combined with metformin, an inhibitor of the mitochondrial NADH dehydrogenase, had interesting anti-cancer properties in HL60 (promyelocytic leukemia), OPM2 (multiple myeloma) and HT1080 (fibrosarcoma) cells. They reported that the association between syrosingopine and metformin was able to induce the loss of NAD^+^ regeneration capacity in tumor cells, which is required for the ATP-generating steps of glycolysis. This resulted in a glycolysis blockade, leading to ATP depletion and cell death.

In the present study, our goal was to characterize the effects of syrosingopine alone as an MCT1 and MCT4 inhibitor on different metabolic processes in vitro, including extracellular acidification rate (ECAR), glucose metabolism, intra- and extra-cellular pH (pHi and pHe, respectively), cell proliferation and viability. As we could anticipate differential effects depending on the metabolic phenotypes, these effects were evaluated in the MDA-MB-231 glycolytic breast cancer model and in the oxidative FaDu squamous cell carcinoma of the hypopharynx [15,19,20]. As effects were more important in the breast cancer model, the impact of syrosingopine on pHe was also evaluated in vivo in the MDA-MB-231 tumor model, using non-invasive Chemical Exchange Saturation Transfer (CEST)-MRI [3,21,22].

## 2. Results

### 2.1. MCT1 and MCT4 Expressions in MDA-MB-231 and FaDu Cells

**Figure 1:**
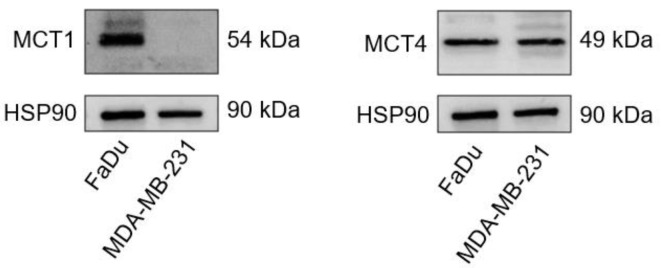
Western blot of MCT1 and MCT4 in FaDu and MDA-MB-231 cells. HSP90 was used as loading control.

The basal expressions of MCT1 and MCT4 were evaluated in MDA-MB-231 and FaDu cells. We observed that MCT4 was expressed in both cell lines, while MCT1 was only expressed in FaDu cells (Figure 1). These results are in accordance with a study conducted by Hussien in 2011, where they showed that MCT1 is silenced by gene methylation in human breast cancer cell lines [23].

### 2.2. Exposure to Syrosingopine Reduces the Extracellular Acidification Rate and Induces Intracellular Acidification in Cancer Cells

The influence of treatment with syrosingopine on the extracellular acidification rate (ECAR) and intracellular acidification was first assessed.

**Figure 2:**
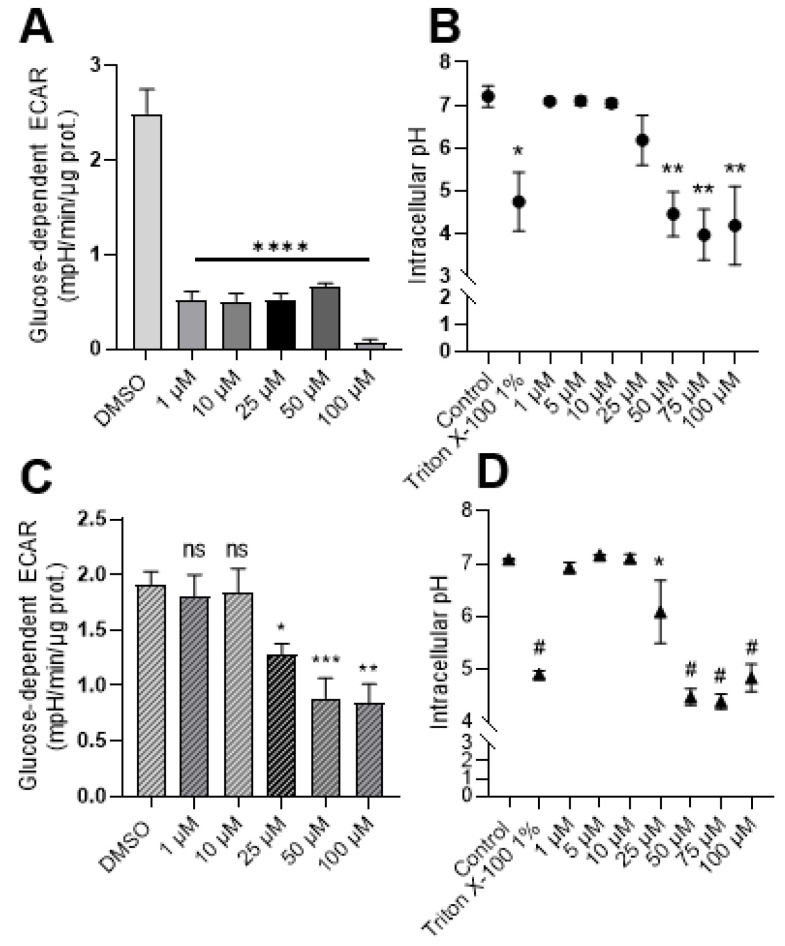
Syrosingopine reduces the extracellular acidification rate (ECAR) and decreases intracellular pH in cancer cells. Glucose-dependent ECAR was determined using the Seahorse XF96 bioenergetic analyzer in MDA-MB-231 (**A**) and FaDu cells (**C**) after 24 h of treatment with increasing concentrations of syrosingopine. Intracellular pH values were measured in MDA-MB-231 (**B**) and FaDu cells (**D**) after 72 h of treatment with increasing concentrations of syrosingopine. Data are represented as mean ± SEM of at least three independent experiments. Significance was determined using one-way or two-way ANOVA: * *p* < 0.05; ** *p* < 0.01; *** *p* < 0.001; # = **** *p* < 0.0001. ns = not significant.

As a consequence of MCT inhibition, we found that exposure to increasing concentrations of syrosingopine induced a significant reduction in the extracellular acidification rate (ECAR) in MDA-MB-231 cells (Figure 2A). The decrease in ECAR was stable over time when the experiments were repeated with cells treated with 10 µM, with no difference between an exposition of 24 or 72 h (data not shown). For the more oxidative FaDu model, a significant decrease in ECAR was observed after treatment with 25 µM of syrosingopine (Figure 2C). No differences were observed when FaDu cells were treated for 24–48 or 72 h with 10 µM of syrosingopine (data not shown). Intracellular pH values were not impacted significantly after 24 h of treatment (data not shown). Reduced pHi values were observed, starting at 50 µM for MDA-MB-231 cells and 25 µM for FaDu when cells were treated for 72 h (Figure 2B–D).

We found that syrosingopine had an impact on ECAR after 24 h, while changes in intracellular pH values were observed only after 72 h.

### 2.3. Metabolic Shifts after Exposure to Syrosingopine and Consequences on Proliferation and Viability

To evaluate the impact of changes in intra- and extra-cellular pH, we analyzed the effect of syrosingopine on glucose metabolism, cell proliferation, cell viability and apoptosis.

**Figure 3:**
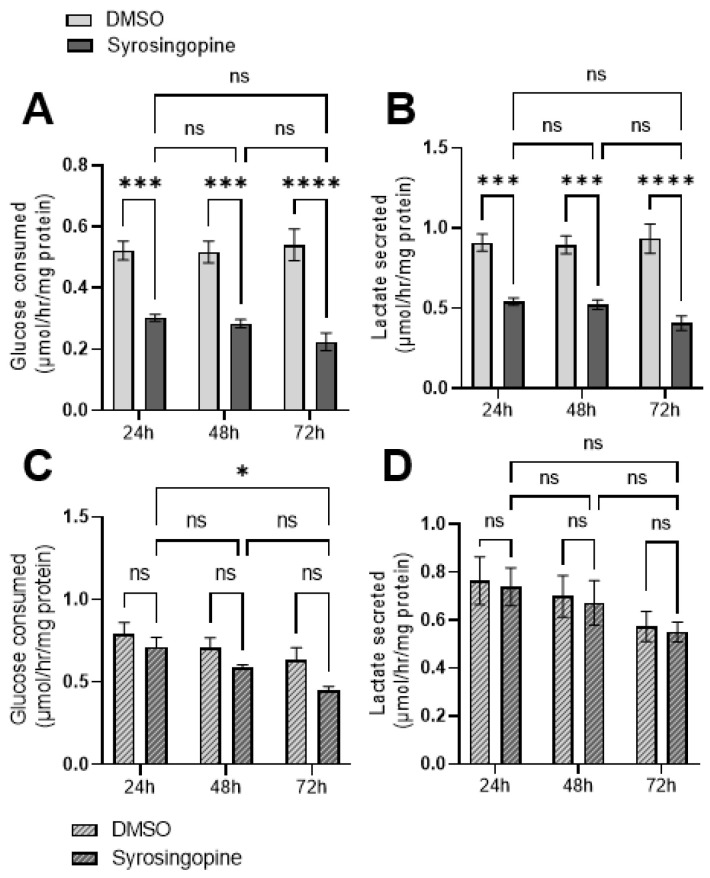
Impact of syrosingopine on glucose consumption and lactate secretion. Glucose consumption and lactate secretion in MDA-MB-231 (**A**,**B**) and FaDu (**C**,**D**) cell lines. Cells were treated for 24, 48 and 72 h with 10 µM of syrosingopine. Data are represented as mean ± SEM of at least three independent experiments. Significance was determined using two-way ANOVA: * *p* < 0.05; *** *p* < 0.001; **** *p* < 0.0001. ns = not significant.

Glucose consumption and lactate secretion were assessed upon direct measurements of metabolite concentrations in the extracellular medium of FaDu and MDA-MB-231 cancer cells treated for 24, 48 and 72 h with 10 µM syrosingopine. Significant reductions in glucose consumption and lactate secretion were observed in MDA-MB-231 (Figure 3A,B) but not in FaDu cells (Figure 3C,D).

**Figure 4:**
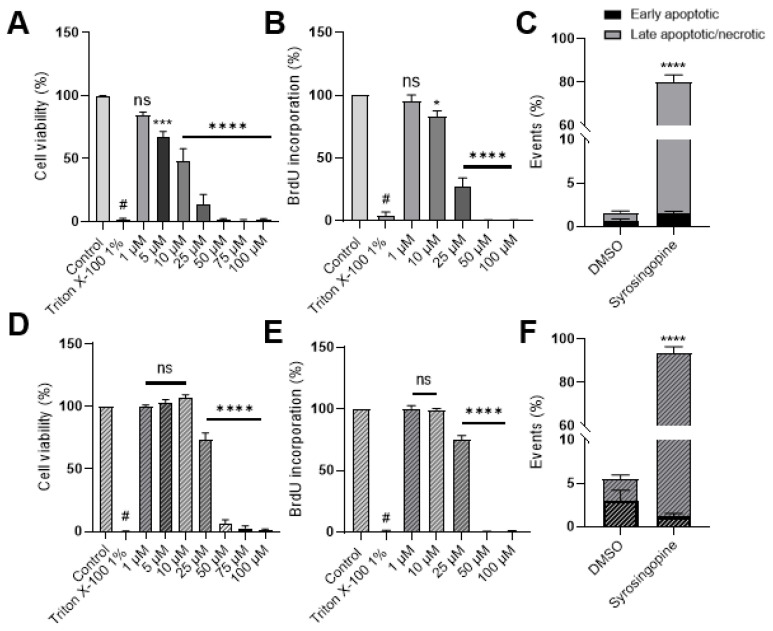
Impact of syrosingopine on tumor cell viability and proliferation. MDA-MB-231 (top, **A**–**C**) and FaDu (bottom, **D**–**F**) cells were treated with syrosingopine for 24, 48 and 72 h. Cell viability (**A**,**D**) and proliferation (**B**,**E**) are represented after 72 h of treatment. Percentages of early apoptotic/necrotic cells were calculated after treatment with 25 µM of syrosingopine for 72 h (**C**,**F**). Data are represented as mean ± SEM of at least three independent experiments. Significance was determined using one-way or two-way ANOVA: * *p* < 0.05; *** *p* < 0.001; # = **** *p* < 0.0001. ns = not significant.

Cell proliferation was assessed using a bromodeoxyuridine (BrdU) proliferation assay. BrdU is a synthetic analogue of thymidine, which can be incorporated into cells during the S phase of the cell cycle. We observed that proliferation was significantly reduced when cells were treated for 72 h with increasing concentrations of syrosingopine, starting at 5 µM for MDA-MB-231 and 25 µM for FaDu cells (Figure 4A–D).

A Presto Blue assay was used to determine the effect on cell viability. This test is based on the reduction of the weakly fluorescent dye resazurin to highly fluorescent resorufin by cell mitochondria. Cell viability can thus be extrapolated from the fluorescence signal. Viability was impacted, starting at a concentration of 5 µM syrosingopine for MDA-MB-231 and 25 µM for FaDu cells (Figure 4B–E).

Fluorescence-Activated Cell Sorting (FACS) using propidium iodine and Annexin V was performed at 10 µM and 25 µM. Treatment with 10 µM for 72 h did not affect cell mortality, as we did not observe any difference between control or treated cells (data not shown). However, when cells were treated with 25 µM, an increase in the percentage of late apoptotic and/or necrotic cells was noticed, after 72 h (Figure 4C–F).

Syrosingopine impacts glucose metabolism, as well as cell viability, cell proliferation and apoptosis.

### 2.4. In Vivo pHe Measurements with CEST-MRI

**Figure 5:**
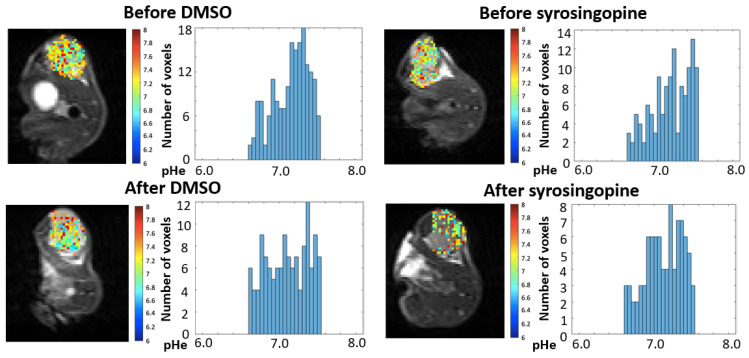
Impact of syrosingopine on tumor pHe. The evolution of pHe was evaluated using CEST-MRI in MDA-MB-231 tumor xenografts. Typical images and histograms of distribution of pHe values recorded before and after vehicle administration (**left**), and before and after syrosingopine administration (**right**) (daily IP injection over 4 days, 5 mg/kg). Images were acquired from the same mice.

As the effects of syrosingopine on tumor cell proliferation were more pronounced on MDA-MB-231 cells than on FaDu cells, in vivo studies were carried out on the MDA-MB-231 tumor model to measure potential changes in pHe and in tumor growth. CEST-MRI was applied using a protocol adapted from our previous work on mitochondrial pyruvate carrier (MPC) inhibition [24]. The administration pattern of syrosingopine was inspired by the work of Benjamin et al. [17,18]. The MR protocols were applied before and after 4 days of treatment with syrosingopine (5 mg/kg body weight, daily administration) or vehicle (DMSO). The distribution of pHe values before and after treatment is presented in Figure 5. Ten tumors were analyzed in each group. Acquisitions were acquired before and after the injection of iopamidol, used as a pH-sensitive CEST contrast agent.

**Figure 6:**
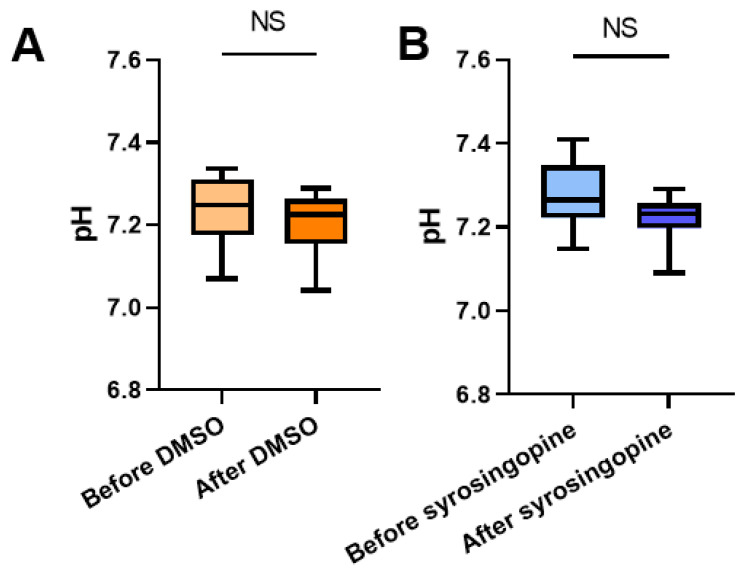
Impact of syrosingopine on tumor pHe. Evolution of pHe (estimated using CEST-MRI) in MDA-MB-231 tumors in mice before and after 4 days of treatment with DMSO (control, (**A**)) or syrosingopine (**B**). Data are represented as mean ± SEM. Significance was determined using Paired *t*-test; *n* = 10 in both groups. NS = not significant.

**Figure 7:**
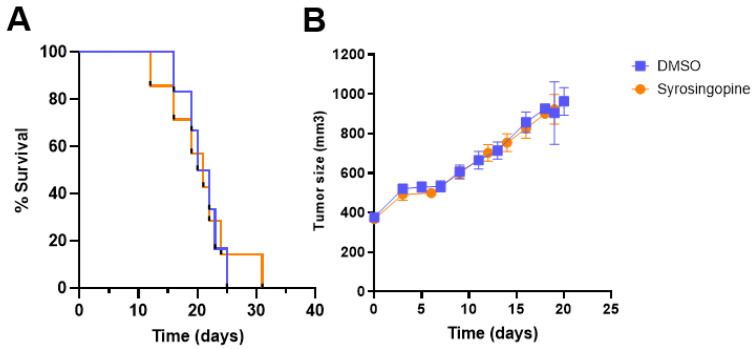
Impact of syrosingopine treatment on tumor growth and survival. (**A**): Evolution of tumor size over time in control (blue) and syrosingopine-treated mice (daily IP injection over 4 days, 5 mg/kg) (orange). (**B**): percentage of survival of mice after treatment with DMSO (control) or syrosingopine for 4 days. Data are represented as mean ± SEM; *n* = 10 in both groups.

We did not observe any significant difference in the mean or median values between the treated and control groups (Figure 6). The histograms of the pHe distribution show that the pHe values are similar before and after treatment in both groups. Besides the MRI analysis, we studied the effects of treatment with syrosingopine on tumor growth. We did not observe different growth patterns between the groups (Figure 7A). There was also no difference in survival between the two groups (Figure 7B).

## 3. Discussion

Targeting proton extrusion in cancer represents a promising therapeutic approach. Being able to monitor the effect of treatments on tumor growth and therapeutic response using noninvasive magnetic resonance methods could be a step forward to personalized medicine. In this work, we focused on an MCT1/4 inhibitor, syrosingopine, and its effects on several metabolic processes and on extracellular acidification, in vitro and in vivo. First, we showed that the inhibition of MCT with syrosingopine in vitro reduced extracellular acidification in two different cell types: breast cancer MDA-MB-231 and pharynx squamous cell carcinoma FaDu cells, after 24 h of treatment. Discrepancies between the cell lines could be due to their different metabolic profiles. MDA-MB-231 cells are well known to have a more glycolytic metabolism, while FaDu cells tend to depend more on oxidative respiration [15,19,20]. We speculate that MDA-MB-231 cells are more sensitive to the inhibition of MCT because they rely more on glucose metabolism.

As MCT1 is not expressed in MDA-MB-231 cells, the effect observed is likely due to MCT4 inhibition. MCT4 is responsible for the excretion of lactate out of cells, with protons. Treatment with syrosingopine should lead to an accumulation of lactate inside cells together with a drop in glucose consumption, intracellular acidification and a reduction in lactate secretion. Our observations are consistent with this scenario in the glycolytic MDA-MB-231 tumor model. We also noted a reduction in cell proliferation and an increase in the number of late apoptotic/necrotic cells when they were treated with 25 µM syrosingopine. The effects on glucose and lactate were less pronounced in FaDu cells, but the effects on intra- and extra-cellular pH values, cell proliferation, viability and apoptosis were still notable. Altogether, these observations support the idea that the co-expression of MCT1 and MCT4 in the same cancer cell (as reported in FaDu cells) may facilitate their metabolic plasticity by adapting their bioenergetics and/or biosynthetic preferences to grow and survive under drug- and/or environment-induced stress. In line with this, a recent study has reported that cancer cells become highly dependent on MCT4 expression/activity when MCT1 expression is epigenetically lost upon exposure with the anti-metabolic drug 3-bromopyruvate [25], thereby highlighting the need to identify the best combination of treatments to overcome tumor metabolic plasticity [26,27].

In light of all these in vitro results, syrosingopine was considered for in vivo studies. Syrosingopine has been reported to induce the sensitization of tumors to metformin, an antidiabetic and mitochondrial inhibitor, in vivo. Benjamin et al. observed a reduction in the number of tumor nodules when mice were treated with either one or a combination of the two molecules [17]. We decided to test the effect of chronic treatment with syrosingopine alone on extracellular pH in MDA-MB-231 tumors. As it is an antihypertensive drug, we selected a lower dose than Benjamin et al. did, as our treatment period was longer, and we wanted to avoid any hemodynamic consequences that could change tumor perfusion. The 4-day treatment did not affect either extracellular pH, tumor growth or mice mortality.

The discrepancies between the in vitro and in vivo results could be explained in several ways. The first hypothesis is that the dose that we used was not sufficient to mimic the actual dose used in vitro. The amount of syrosingopine that actually reached the tumor was not sufficient to recapitulate the concentration used in vitro. Unfortunately, dose escalation over 5 mg/kg was precluded, as syrosingopine presents hypotensive effects. It was indeed previously described that syrosingopine deprived rodents from sympathetic vasomotor tone by the depletion of peripheral neuronal noradrenaline stores at a similar dose (5 mg/kg) to the one used in our study [28]. The absence of effects could also be due to the fact that the period of treatment was not long enough to induce changes in pHe. In our study, mice were treated with syrosingopine or vehicle (DMSO) every day for 4 days in total, whereas Benjamin et al. treated their mice every two days for 16 days. We cannot exclude that a longer period of treatment could affect pHe values, as well as tumor growth. Another possibility is that syrosingopine can only induce little to no effects when used on its own. Syrosingopine is known to inhibit both MCT1 and MCT4, but it is around 60-fold more potent against MCT4 than MCT1 and only at high concentrations. Further studies are needed to evaluate the effect of MCT inhibition in vivo, with more potent inhibitors and with Ki reported in the nM range in order to avoid off-target effects.

## 4. Materials and Methods

### 4.1. Cell Culture

MDA-MB-231 cells were cultured at 37 °C in a humidified atmosphere with 5% CO_2_ and maintained in DMEM medium (Thermo Fisher Scientific, Merelbeke, Belgium) supplemented with 10% heat-inactivated FBS. FaDu cells were maintained in DMEM GlutaMAX medium supplemented with 10% FBS and 1% non-essential amino acids. Both cell lines were acquired from ATCC, where they are regularly authenticated by short tandem repeat profiling. All cell lines were tested for mycoplasma contamination with the PCR-based MycoplasmaCheck assay (Eurofins, Ebersberg, Germany) before being used.

### 4.2. Glucose Consumption, Lactate Secretion and Extracellular Acidification Rate (ECAR)

For glucose and lactate dosages, cells (2 × 10^5^ cells/well; 3 wells/condition) were seeded in 12-well plates with 2 mL of their routine culture medium. After 24 h, medium was replaced by 500 µL of DMEM containing 10 mmol/L D-glucose and supplemented with 2 mmol/L L-glutamine and 10% dialyzed FBS (Sigma-Aldrich, Overijse, Belgium), in the presence of 10 µmol/L syrosingopine (Sigma-Aldrich, Overijse, Belgium) or vehicle (DMSO; Sigma- Aldrich, Overijse, Belgium). The initial concentrations of glucose and lactate in the experimental medium were also assessed by including control wells containing only cell culture medium (no cells) on each plate. After incubation for 24 h, extracellular media were collected and deproteinized by centrifugation (15 min, 10,000 rpm, 4 °C) in 10 kDa cut-off filter tubes (VWR, Leuven, Belgium). Glucose and lactate concentrations were measured in the samples (50 µL) using enzymatic assays (CMA Microdialysis AB, Kista, Sweden) and a CMA 600 analyzer (Aurora Borealis, Solna, Sweden). Data analysis was carried out by calculating the difference in metabolite concentrations between the control wells and the experimental wells. Data were then normalized by the protein content in each well and expressed in µmol/h/mg protein.

Extracellular acidification rates (ECARs) were measured using the Seahorse XF96 analyzer (Agilent, Santa Clara, CA, USA). Briefly, cells (1.5 × 10^4^ cells/well; 6 wells/condition) were seeded in Seahorse 96-well cell culture plates in their routine culture medium, in the presence of different concentrations of syrosingopine or vehicle (DMSO). After 24 h, medium was replaced by 175 µL unbuffered serum-free DMEM, pH 7.4, supplemented with 2 mmol/L L-glutamine, still in the presence of the treatment. ECAR values were assessed before and after the injection of 10 mmol/L D-glucose. Glucose-dependent ECAR was calculated by comparing the values before and after addition of the substrate. Data were normalized by the protein content in each well and expressed in mpH/min/µg protein.

### 4.3. Western Blot

Adherent MDA-MB-231 and FaDu cells were lysed in RIPA buffer (Thermo Scientific, Merelbeke, Belgium) supplemented with 1% protease and phosphatase inhibitors (Thermo Scientific, Merelbeke, Belgium). Protein amount in whole-cell lysates was measured with a Pierce™ BCA Protein Assay Kit (Thermo Scientific, Merelbeke, Belgium). Equal amounts of protein were loaded onto 4–15% Mini-PROTEAN^®®^ TGX™ Precast Gels (Bio-Rad, Temse, Belgium). Following electrophoresis in 1x Tris/glycine/SDS running buffer (Bio-Rad, Temse, Belgium), proteins were transferred to PVDF membranes using the Trans-Blot^®®^ Turbo™ RTA Mini PVDF Transfer Kit (Bio-Rad, Temse, Belgium) according to the manufacturer’s instructions. Non-specific binding was blocked by soaking the membranes in 5% milk in tTBS (1x Tris-Buffered Saline, 0.1% Tween 20, Bio-Rad, Temse, Belgium) at room temperature for 1 h. Membranes were incubated with primary anti-HSP90 (4875S, Cell Signaling, Leiden, The Netherland), anti-MCT1 (Ab93048, Abcam, Amsterdam, The Netherland) and anti-MCT4 (AB3316P, Sigma-Aldrich, Hoeilaart, Belgium) antibodies in tTBS/milk 5% at 4 °C overnight, followed by incubation with anti-rabbit or anti-mouse secondary antibodies (Jackson IR, Ely, UK) in tTBS/milk 1% at room temperature for 1 h. Detection was performed using the SuperSignal™ West Pico Plus kit (Thermo Scientific, Merelbeke, Belgium).

### 4.4. Cell Viability

Cell viability was assessed using the Presto Blue reagent (Thermo Scientific, Merelbeke, Belgium) according to the manufacturer’s instructions. MDA-MB-231 (7000 cells/well) or FaDu cells (5000 cells/well) were seeded in 96-well plates. Increasing concentrations of syrosingopine were tested, and cells were incubated for 24, 48 or 72 h. Cell growth was measured by incubating the cells with 10% Presto Blue reagent for 1 h. Fluorescence was measured at 560 nm after excitation at 590 nm with a SpectraMax plate reader (M2 Molecular Devices, Wokingham, UK).

### 4.5. Cell Proliferation Assay

To assess cell proliferation, a 5-bromo-2′-deoxyuridine (BrdU) assay was performed using a BrdU Cell Proliferation Assay Kit according to the manufacturer’s instructions (Sigma Aldrich, Overijse, Belgium). MDA-MB-231 and FaDu cells were seeded at 7000 and 5000 cells/well, respectively, in a 96-well plate and incubated overnight. To assess proliferative activity under different concentrations of syrosingopine, cells were incubated for 24, 48 or 72 h. On the day of the experiment, 20 µL of BrdU was added to each well, and cells were incubated for 2 h. The absorbance at a wavelength of 370 and 492 nm was measured using a SpectraMax (M2 Molecular Devices, Wokingham, UK).

### 4.6. Intracellular pH Measurements

Intracellular pH was measured using the pH sensor 5-(and-6)-carboxy-seminaphthorhodafluor-acetoxymethylester (C.SNARF1-AM; Invitrogen, Merelbeke, Belgium). Cells were seeded in a 96-well plate. Calibration solutions (pH 6.6, 6.8, 7, 7.2, 7.4 and 8) were prepared in order to calculate pHi values. On the day of the analysis, cells were incubated with pH-sensitive C-SNARF1-AM for 30 min. After washing, fluorescence was measured at 580 and 642 nm after excitation at 485 nm using a SpectraMax (M2 Molecular Devices, Wokingham, UK).

### 4.7. Apoptosis/Necrosis

Apoptotic changes in cells were assessed using the eBioscience ™ Annexin V Apoptosis Detection Kit APC (Sigma Aldrich, Overijse, Belgium). A total of 80,000 MDA-MB-231 and 70,000 FaDu cells per condition were plated in a 6-well plate 4 days before the experiment. Cells were treated for 24 h, 48 h or 72 h before the experiment with 10 or 25 µM of syrosingopine or with DMSO into their respective culture media. On the day of the experiment, cells were detached with trypsin (0.05%), harvested into their respective old media, transferred into a FACS tube and centrifuged. The staining protocol briefly included washing the cells with PBS and then adding 80 μL of binding buffer along with 5 μL Annexin V (AnnV) and 5 μL propidium iodide (PI) per tube. Cells were kept in the dark for 15 min until FACS assessment, with an additional 100 μL of binding buffer. Data were acquired using a BD FACSCantoII flow cytometer equipped with Diva Software (BD Biosciences, Erembodegem, Belgium) and processed with FlowJo software (BD Biosciences, Erembodegem, Belgium). At least 10,000 events were analyzed for each condition. Cells’ subpopulations were identified according to unstained control cells and distinguished between alive (AnV− PI−), early apoptotic (AnV+ PI−) and late apoptotic/necrotic (AnV+ PI+).

### 4.8. Tumor Model

All experiments involving animals were performed in accordance with the Belgian law concerning the protection and welfare of animals and were approved by the UCLouvain Ethics Committee (Agreement reference: 2018/UCL/MD/021).

MDA-MB-231 cells (10 × 10^6^ cells in 100 µL of PBS) were injected intramuscularly in the right hind paw of 6-week-old female NMRI nude mice (Janvier). When tumors reached a volume of ± 350 mm^3^, the pHe of tumors was measured using CEST-MRI. Mice were treated with a daily intraperitoneal injection of syrosingopine (5 mg/kg) or vehicle for 4 days. The pHe was measured after treatment. Tumor growth was monitored until tumors reached a size of ± 1500 mm^3^. Tumor size were monitored three times per week using an electronic caliper, and two distances were measured, X and Y (X < Y). Tumor shape was assumed to be ellipsoidal; hence, the volume was considered Pi/6.

### 4.9. CEST-MRI Experiments

#### 4.9.1. CEST Calibration In Vitro

All experiments were performed on an 11.7T Biospec MRI (Bruker, Ettlingen, Germany). Calibration studies were performed as previously described [24]. CEST acquisitions were acquired with a ^1^H volume coil with a 40 mm inner diameter. The CEST sequence was a RARE sequence (TR = 4.0 s, TE = 23.0 milliseconds, rare factor = 10, slice thickness = 2 mm, FOV = 30 mm, matrix size = 64 × 64, in-plane special resolution = 468 µm) with an additional continuous 4 µT irradiation for 4 s. The Z spectrum was acquired between 8 and 8 ppm (81 frequencies) for a total acquisition time of 27 min 20 s.

#### 4.9.2. In Vivo Analysis

In vivo studies were performed as described in [24], but a modified version of the CEST protocol was applied. First, acquisitions were acquired before the injection of iopamidol. CEST images were acquired using the same parameters as in the in vitro experiment. Iopamidol was then injected intravenously through the tail vein (3 g Iodine/kg). The second acquisition started 20 min after the injection. All voxels were analyzed using a custom-written script in Matlab (The MathWorks Inc., Natick, MA, USA). The Z-spectra were centered on the bulk water signal to correspond to the zero frequency. Values were measured at the frequency offsets of 4.2 and 5.5 ppm and used in Equation (1):

ST = (S_−Δω_ − S_+Δω_)/S_0_ (1)

where S = signal intensity at 4.2 or 5.5 ppm, and S0 = bulk water signal intensity without saturation. Saturation transfer values at 4.2 and 5.5 ppm can be used to calculate their ratio (2):

RST = (ST 5.5)/(ST 4.2) (2)

In order to eliminate outliers, different filters were applied to the data. A threshold of 2% was used to discriminate between enhanced and non-enhanced voxels. Negative ST values were excluded, as well as RST values above 0.2 and below 1.55. pH values were calculated based on the RST and the corresponding pH values of the calibration curve. pH maps were obtained by overlaying anatomical images and pH values in each voxel. Only tumors with a pH map that included more than 10 voxels (representing a total volume of 4.4 µL) were kept for the statistical analysis.

### 4.10. Statistical Analysis

Statistical analysis was performed using GraphPad Prism software (San Diego, CA, USA). Significance was determined using one-way or two-way ANOVA. In vivo pH values before and after treatments were compared using a paired *t*-Student test, with *p* ≤ 0.05 considered significant. Results are represented as mean ± SEM. The number of experiments is provided in each figure legend.

## 5. Conclusions

The inhibition of MCT with syrosingopine led to large effects in vitro on several important metabolic processes in MDA-MB-231 and FaDu tumor cell models. In vivo, no significant changes in pHe values, tumor growth or survival were observed, as we were limited by the potential off-target effects of syrosingopine on hemodynamics. Our study shows that MCT represents an interesting target for anti-cancer therapies but that further studies with more specific MCT inhibitors are needed to determine the effect of its inhibition in vivo.

## Data Availability

Data is contained within the article.

## References

[1] Hanahan D., Weinberg R.A. (2011). Hallmarks of cancer: The next generation. Cell.

[2] Fang J.S., Gillies R.D., Gatenby R.A. (2008). Adaptation to hypoxia and acidosis in carcinogenesis and tumor progression. Semin. Cancer Biol..

[3] Anemone A., Consolino L., Arena F., Capozza M., Longo D.L. (2019). Imaging tumor acidosis: A survey of the available techniques for mapping in vivo tumor pH. Cancer Metastasis Rev..

[4] Gatenby R.A., Gillies R.J. (2004). Why do cancers have high aerobic glycolysis?. Nat. Rev. Cancer.

[5] Wojtkowiak J.W., Verduzco D., Schramm K.J., Gillies R.J. (2011). Drug resistance and cellular adaptation to tumor acidic pH microenvironment. Mol. Pharm..

[6] Linden C.V., Corbet C. (2019). Therapeutic Targeting of Cancer Stem Cells: Integrating and Exploiting the Acidic Niche. Front. Oncol..

[7] Corbet C., Feron O. (2017). Tumour acidosis: From the passenger to the driver’s seat. Nat. Rev. Cancer.

[8] Neri D., Supuran C.T. (2011). Interfering with pH regulation in tumours as a therapeutic strategy. Nat. Rev. Drug Discov..

[9] Pillai S.R., Damaghi M., Marunaka Y., Spugnini E.P., Fais S., Gillies R.J. (2019). Causes, consequences, and therapy of tumors acidosis. Cancer Metastasis Rev..

[10] De Milito A., Iessi E., Logozzi M., Lozupone F., Spada M., Marino M.L., Federici C., Perdicchio M., Matarrese P., Lugini L. (2007). Proton pump inhibitors induce apoptosis of human B-cell tumors through a caspase-independent mechanism involving reactive oxygen species. Cancer Res..

[11] Taylor S., Spugnini E.P., Assaraf Y.G., Azzarito T., Rauch C., Fais S. (2015). Microenvironment acidity as a major determinant of tumor chemoresistance: Proton pump inhibitors (PPIs) as a novel therapeutic approach. Drug Resist. Updates.

[12] Dolatkhah M., Omidi Y. (2019). Renewed interests in carbonic anhydrase IX in relevance to breast cancer treatment. Bioimpacts.

[13] Pinheiro C., Longatto-Filho A., Azevedo-Silva J., Casal M., Schmitt F.C., Baltazar F. (2012). Role of monocarboxylate transporters in human cancers: State of the art. J. Bioenerg. Biomembr..

[14] Counillon L., Bouret Y., Marchiq I., Pouyssegur J. (2016). Na(+)/H(+) antiporter (NHE1) and lactate/H(+) symporters (MCTs) in pH homeostasis and cancer metabolism. Biochim. Biophys. Acta.

[15] Sonveaux P., Vegran F., Schroeder T., Wergin M.C., Verrax J., Rabbani Z.N., De Saedeleer C.J., Kennedy K.M., Diepart C., Jordan B.F. (2008). Targeting lactate-fueled respiration selectively kills hypoxic tumor cells in mice. J. Clin. Investig..

[16] Le Floch R., Chiche J., Marchiq I., Naiken T., Ilc K., Murray C.M., Critchlow S.E., Roux D., Simon M.P., Pouysségur J. (2011). CD147 subunit of lactate/H^+^ symporters MCT1 and hypoxia-inducible MCT4 is critical for energetics and growth of glycolytic tumors. Proc. Natl. Acad. Sci. USA.

[17] Benjamin D., Colombi M., Hindupur S.K., Betz C., Lane H.A., El-Shemerly M.Y., Lu M., Quagliata L., Terracciano L., Moes S. (2016). Syrosingopine sensitizes cancer cells to killing by metformin. Sci. Adv..

[18] Benjamin D., Robay D., Hindupur S.K., Pohlmann J., Colombi M., El-Shemerly M.Y., Maira S.M., Moroni C., Lane H.A., Hall M.N. (2018). Dual Inhibition of the Lactate Transporters MCT1 and MCT4 Is Synthetic Lethal with Metformin due to NAD+ Depletion in Cancer Cells. Cell Rep..

[19] De Preter G., Danhier P., Porporato P.E., Payen V.L., Jordan B.F., Sonveaux P., Gallez B. (2016). Direct Evidence of the Link Between Energetic Metabolism and Proliferation Capacity of Cancer Cells In Vitro. Adv. Exp. Med. Biol..

[20] Corbet C., Bastien E., Draoui N., Doix B., Mignion L., Jordan B.F., Marchand A., Vanherck J.C., Chaltin P., Schakman O. (2018). Interruption of lactate uptake by inhibiting mitochondrial pyruvate transport unravels direct antitumor and radiosensitizing effects. Nat. Commun..

[21] Consolino L., Anemone A., Capozza M., Carella A., Irrera P., Corrado A., Dhakan C., Bracesco M., Longo D.L. (2020). Non-invasive Investigation of Tumor Metabolism and Acidosis by MRI-CEST Imaging. Front. Oncol..

[22] Chen L.Q., Pagel M.D. (2015). Evaluating pH in the Extracellular Tumor Microenvironment Using CEST MRI and Other Imaging Methods. Adv. Radiol..

[23] Hussien R., Brooks G.A. (2011). Mitochondrial and plasma membrane lactate transporter and lactate dehydrogenase isoform expression in breast cancer cell lines. Physiol. Genom..

[24] Buyse C., Joudiou N., Corbet C., Feron O., Mignion L., Flament J., Gallez B. (2021). Impact of Inhibition of the Mitochondrial Pyruvate Carrier on the Tumor Extracellular pH as Measured by CEST-MRI. Cancers.

[25] Vander Linden C., Corbet C., Bastien E., Martherus R., Guilbaud C., Petit L., Wauthier L., Loriot A., De Smet C., Feron O. (2021). Therapy-induced DNA methylation inactivates MCT1 and renders tumor cells vulnerable to MCT4 inhibition. Cell Rep..

[26] Oshima N., Ishida R., Kishimoto S., Beebe K., Brender J.R., Yamamoto K., Urban D., Rai G., Johnson M.S., Benavides G. (2020). Dynamic Imaging of LDH Inhibition in Tumors Reveals Rapid In Vivo Metabolic Rewiring and Vulnerability to Combination Therapy. Cell Rep..

[27] Beloueche-Babari M., Wantuch S., Galobart T.C., Koniordou M., Parkes H.G., Arunan V., Chung Y.L., Eykyn T.R., Smith P.D., Leach M.O. (2017). MCT1 Inhibitor AZD3965 Increases Mitochondrial Metabolism, Facilitating Combination Therapy and Noninvasive Magnetic Resonance Spectroscopy. Cancer Res..

[28] Lefevre-Borg F., Cavero I. (1980). Stimulation of peripheral dopamine receptors in rats: A mechanism for novel antihypertensive agents. Clin. Sci..

